# Re-construction of action awareness depends on an internal model of action-outcome timing

**DOI:** 10.1016/j.concog.2014.01.007

**Published:** 2014-04

**Authors:** Max-Philipp Stenner, Markus Bauer, Judith Machts, Hans-Jochen Heinze, Patrick Haggard, Raymond J. Dolan

**Affiliations:** a Wellcome Trust Centre for Neuroimaging, University College London, London WC1N 3BG, UK; b Department of Neurology, University of Magdeburg, 39120 Magdeburg, Germany; c Institute of Cognitive Neuroscience, University College London, London WC1N 3AR, UK; d German Centre for Neurodegenerative Diseases, 39120 Magdeburg, Germany

**Keywords:** Action awareness, Temporal binding, Sense of agency

## Abstract

The subjective time of an instrumental action is shifted towards its outcome. This temporal binding effect is partially retrospective, i.e., occurs upon outcome perception. Retrospective binding is thought to reflect post-hoc inference on agency based on sensory evidence of the action – outcome association. However, many previous binding paradigms cannot exclude the possibility that retrospective binding results from bottom-up interference of sensory outcome processing with action awareness and is functionally unrelated to the processing of the action – outcome association. Here, we keep bottom-up interference constant and use a contextual manipulation instead. We demonstrate a shift of subjective action time by its outcome in a context of variable outcome timing. Crucially, this shift is absent when there is no such variability. Thus, retrospective action binding reflects a context-dependent, model-based phenomenon. Such top-down re-construction of action awareness seems to bias agency attribution when outcome predictability is low.

## Highlights

• Action awareness can shift retrospectively at the time of an action-outcome.
• In principle, this could reflect bottom-up interference and not subjective agency.
• We keep bottom-up drive constant and manipulate temporal outcome variability instead.
• Action awareness is shifted retrospectively in a context of variable outcome timing.
• This top-down process may bias subjective agency when an outcome is unpredictable.

## 1 Introduction

When a motor action is followed by a sensory event within a few hundred milliseconds, the subjective time of the action can be shifted towards that sensory event (Haggard, Clark, & Kalogeras, 2002; for a review, see Moore & Obhi, 2012). This binding of subjective action time by a subsequent stimulus depends on learning the underlying contingency (Moore, Lagnado, Deal, & Haggard, 2009; Walsh & Haggard, 2013). It is considered to reflect intentional (Haggard et al., 2002) and/or causative (Buehner & Humphreys, 2009) aspects of the learnt action – outcome association that contribute to a pre-reflective sense of agency (Moore & Obhi, 2012).

Temporal action binding is partially retrospective, in so far as it occurs at the time of outcome perception (Moore & Haggard, 2008). This retrospective component of temporal binding has frequently been regarded as evidence in favour of a post-hoc implicit inference on agency based on sensory evidence, as opposed to a prospective implicit agency ascription during action preparation and/or execution (Moore & Haggard, 2008). This distinction relates to the broader question (Baldwin et al., 2003) of how awareness of prior expectations of an action-outcome (e.g. during unsuccessful trying) on the one hand, and the observation of physical action consequences on the other, are weighted and integrated in the process of sensing intention and causation (Wegner & Wheatley, 1999; Wolpert & Flanagan, 2001).

These interpretations of retrospective action binding assume an underlying inferential, top-down process. In principal, however, a retrospective change of action awareness could be driven by two physiologically distinct mechanisms. A top-down process could re-construct action awareness by integrating prior knowledge and sensory evidence of the action – outcome association. A similar model-based process has been proposed, for example, for an integration of motor predictions and sensory re-afference in comparator models of motor control (Wolpert & Flanagan, 2001). Alternatively, retrospective action binding could reflect bottom-up interference of sensory outcome processing with on-going cognitive processes involved in action awareness, or, at a lower level, with the integration of visual information in the Libet clock paradigm typically deployed in these experiments (Libet, Gleason, Wright, & Pearl, 1983). In this case, the retrospective shift of subjective action time would be solely stimulus-driven, similar to the effects of backward masking on conscious perception, for example (Werner, 1935), and would be independent of an internal model of the action – outcome association.

Previous evidence of retrospective temporal binding comes predominantly from studies that compare subjective action time in the presence and absence of a subsequent stimulus, i.e., two conditions that differ substantially in bottom-up drive (Moore & Haggard, 2008). In these studies, a shift of subjective action time due to outcome presentation reflects retrospective action awareness. Therefore, these studies cannot distinguish between model-based re-construction of action awareness and bottom-up interference with action awareness. Evidence for the idea that a top-down re-constructive process is involved in retrospective binding comes from a study that manipulated the contingency of an outcome on an action, specifically the probability of an outcome given no preceding action (Moore et al., 2009). This study held bottom-up drive constant across trials of interest and used a contextual manipulation instead to demonstrate that retrospective binding depends on an internal representation of *how necessary an action is* for a sensory event.

Similarly, we here used a contextual manipulation while keeping bottom-up influences constant to address the question whether action awareness depends on an internal model of outcome *timing*. Previous studies have shown that binding of an outcome to an action depends on temporal outcome predictability (Haggard et al., 2002). We asked whether retrospective and prospective components of action awareness also reflect temporal outcome predictability.

Moore and Haggard (2008) have previously shown that outcome presentation shifts subjective action time under conditions of low outcome predictability, as achieved by lowering the probability of outcome *occurrence* (the “whether” of the outcome). Here, we tested whether a contextual manipulation of *temporal* outcome predictability leads to a similar shift of subjective action time. In brief, we compared retrospective binding in two conditions that differ in temporal variability of the outcome with respect to the action (the “when” of the outcome) while keeping the structure and timing of trials of interest constant, i.e., bottom-up drive. Any difference in retrospective binding between conditions of variable and fixed outcome timing would reflect an influence of context, rather than any bottom-up interference with action awareness. In addition, Moore and Haggard (2008) demonstrated a shift of action awareness towards an expected outcome even when the outcome was omitted. The authors interpreted this shift as a prospective component of action awareness. To examine effects of temporal outcome predictability on this prospective component, our study introduced an expectation of outcome occurrence by presenting outcomes on 75% of trials in both “variable” and “fixed” outcome timing blocks.

## 2 Methods

### 2.1 Participants

We recruited 16 healthy volunteers (age 23.5 ± 2.78 years (mean ± SD), 10 females; all right-handed) via an online database. All participants gave written informed consent prior to participation with the right to exit the study at any time. The study was approved by the local ethics committee (University College London, UK). Participants received £10 per hour as reimbursement.

### 2.2 Task

Presentation® software (Neurobehavioral Systems, www.neurobs.com) was used to generate all stimuli and to program the paradigm. The task was presented on an LCD screen (vertical refresh rate 60 Hz) on a white background. The experiment was run in a dark and sound-proof room. Participants were seated 75 cm in front of the screen and kept their chin on a chin rest. Sound was presented binaurally at 74 dB SPL via Sennheiser HD 380 pro headphones.

**Fig. 1:**
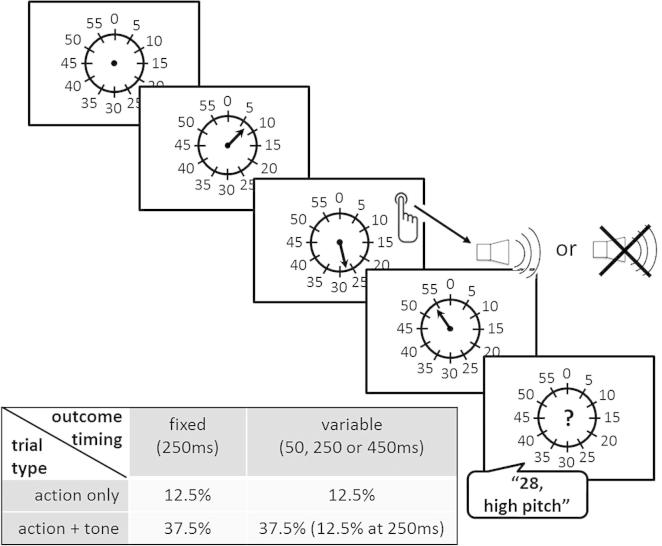
Task design. The structure of one trial and the 2 × 2 factorial study design are shown. Percentages in the table represent the percentage of trials in the two operant (=non-baseline) conditions.

The task (Fig. 1) was a variation of the temporal binding paradigms used by Haggard et al. (2002) and Moore and Haggard (2008). On each trial, participants pressed a button with their right index finger once, at a time of their choice, and verbally reported the time of this button press with respect to the rotating clock hand of a Libet clock (Libet et al., 1983) (period of 2.55 s) at the end of the trial.

The experiment was based on a 2 × 2 factorial design, with one factor (“trial type”, two levels: “action + tone” vs. “action only”) varied on a trial-by-trial basis and the other (“outcome timing variability”: “variable outcome timing” vs. “fixed outcome timing”) varied block-wise. There was an additional, blocked condition that served as baseline for subjective time judgements (“baseline” blocks).

75% of trials in “variable outcome timing” and “fixed outcome timing” blocks were “action + tone” trials, in which participants’ button presses were followed by one of two sine wave tones (750 Hz or 800 Hz, 100 ms duration). Each tone frequency was presented pseudo-randomly in half of these trials. In the remaining 25% of trials, no tone was presented. These trial frequencies of “action + tone” and “action only” trials were identical to Moore and Haggard (2008), where they were used to establish outcome expectation and quantify prospective action binding in trials of unexpected outcome omission. Here, they served to control for potential differences in prospective binding due to “outcome timing variability”: Stronger binding in “action + tone” trials compared to “action only” trials can be attributed to a re-construction triggered by the tone over and above any prospective shift of subjective action time.

Tones followed the button presses either after a fixed delay of 250 ms (in “fixed outcome timing” blocks) or after a variable delay of either 50 ms, 250 ms or 450 ms (with equal probabilities) (in “variable outcome timing” blocks). At the end of “action + tone” trials, participants verbally reported whether the tone they had heard was a high-pitch or low-pitch tone, in addition to reporting the time of their button press. This incidental pitch discrimination task was introduced to prevent participants from ignoring the tones.

There were three “fixed outcome timing” blocks and three “variable outcome timing” blocks, presented consecutively, with the order of the two conditions fully counterbalanced across subjects. Each of these blocks consisted of 24 trials. Outcome timing with respect to the action (fixed or variable) was made explicit at the beginning of each block. In addition, there were two “baseline” blocks, consisting exclusively of “action only” trials. These blocks were presented before and after the main experimental blocks and consisted of 9 trials each. Before the first block in which tones were played, participants were familiarized with the two tone frequencies by listening to them in an alternating sequence for as long as they wanted to.

At the beginning of each trial, the clock hand started to rotate at a random position. Participants were instructed not to press the button during the first rotation of the clock hand. Rotation of the clock hand continued over a total of 350–400 vertical refresh frames and then stopped, largely independently of when participants chose to press the button. Only if participants pressed throughout the last 60 frames before the planned end of rotation, this rotation was extended for 60 more frames after sound presentation (75 frames after the button press in “action only” trials), so that the clock hand never stopped directly or very shortly after the button press. If participants did not press the button at all before the clock hand stopped rotating, a red “x” (font size: 1°) was presented in the centre of the screen, indicating a miss. These trials were repeated at the end of the respective experimental block. During the inter-trial interval, the clock was presented without the clock hand for 1000–1500 ms.

Participants were discouraged from anticipating the clock hand position when pressing the button and from pressing in a stereotyped way. They were asked to fixate a stationary black circle at the centre of the clock at all times, rather than to follow the rotating clock hand with their eyes. Participants were instructed to report the clock hand position only after the clock had stopped rotating and a question mark (font size: 1°) had replaced the clock hand. Lastly, they were asked to be as accurate as possible in their timing reports and to use all integer numbers from 0 to 59, rather than just the multiples of five that were explicitly labelled around the clock face. After verbally reporting their subjective time judgement, the experimenter typed the value into a keyboard.

The image of the clock consisted a circular annulus (outer diameter: 3°, inner diameter: 2.65°), presented at the centre of the screen, twelve radial lines, which represented the five-minute markers (length 0.5°, line width .075°) and which were labelled with the multiples of five between zero and 55 (font size: 0.5°) at 2.15° eccentricity, a central, solid black circle (diameter: 0.3°) and one clock hand (line width: 0.15°, length: 1.4°). All visual elements of the clock were black, the inside of the annulus was white. The clock hand rotated 2.353° upon every vertical refresh (∼17 ms), resulting in one full rotation every 2550 ms. Exact timing of picture stimulus presentation was monitored and trials were repeated at the end of the respective experimental block if at least one picture was delayed by one frame or more.

### 2.3 Data analysis

Our main focus was on participants’ time judgement errors, i.e., the difference between the subjective, reported time of the action and its objective time with respect to the Libet clock. Following previous studies on binding of subjective action time (Haggard et al., 2002), we subtracted the mean time judgement error in “baseline” blocks from mean time judgement errors in “variable outcome timing” and “fixed outcome timing” blocks for each participant. Positive time judgement errors therefore indicate temporal binding of the action by its (expected) outcome. We focussed on subjective time in “action only” trials vs. “action + tone” trials in which event timing was identical in “fixed outcome timing” and “variable outcome timing” blocks, i.e., on “action + tone” trials with an action – outcome delay of 250 ms. In addition, we report results for “action + tone” trials in “variable outcome timing” blocks that were based on the two alternative action – outcome delays separately (50 ms and 450 ms).

To compare attentional demand across conditions, we analysed the variability (standard deviation) of time judgement errors (cf. Haggard et al., 2002) and sensitivity (*d*′) in the incidental pitch discrimination task across participants. A two-way repeated-measures analysis of variances (ANOVA) was used in all cases, followed by pairwise dependent-samples *t*-tests.

## 3 Results

**Fig. 2:**
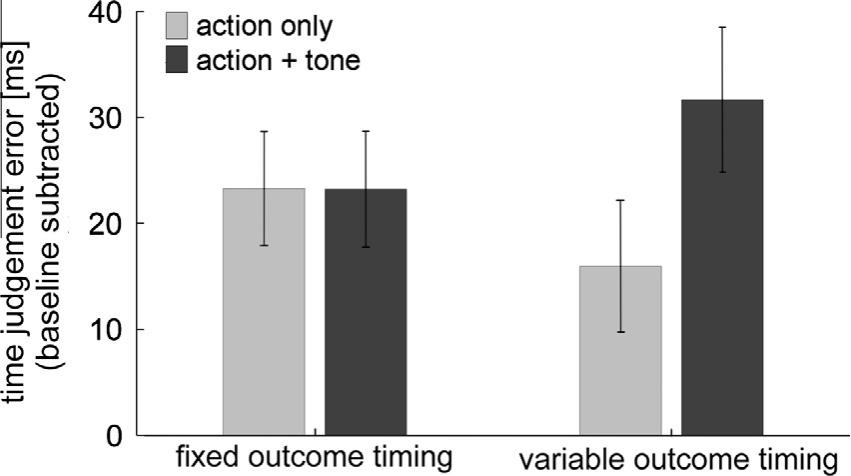
Action binding. Baseline-subtracted mean time judgement errors in the four experimental conditions (mean ± SEM).

We were primarily interested in changes in retrospective binding due to contextual information on outcome timing variability, not due to changes in bottom-up drive. Therefore, we first focused on “action + tone” trials with identical trial event timing in both “variable” and “fixed” outcome timing blocks, i.e., on trials with an action – outcome delay of 250 ms. We compared these “action + tone” trials to “action only” trials in the two types of blocks (Fig. 2).

A two-way repeated-measures ANOVA (“outcome timing variability” × “trial type”) revealed a significant interaction effect (*F*_1,15_ = 6.84, *p* = .019) and a main effect of “trial type” (*F*_1,15_ = 8.5, *p* = .011) on mean time judgement errors across subjects. Post-hoc dependent-samples *t*-tests showed that the interaction was due to a significant shift of subjective action time forwards in “action + tone” trials vs. “action only” trials in “variable outcome timing” blocks (mean ± SD: 31.7 ± 27.4 ms (“action + tone”) vs. 16 ± 24.9 ms (“action only”); *t*_15_ = −3.88, *p* = .001), an effect that was absent in “fixed outcome timing” blocks (mean ± SD: 23.3 ± 21.8 ms (“action + tone”) vs. 23.3 ± 21.5 ms (“action only”); *p* = .99). No other pairwise *t*-test was significant, suggesting that the observed main effect of “trial type” was predominantly driven by the difference between “action only” trials and “action + tone” trials in the ”variable outcome timing” block.

Our paradigm required attention to the pitch of the tone in order to prevent participants from ignoring the tone. In order to test for potential differences in attention towards the tone across conditions, we analysed the standard deviation of time judgement errors, which has previously been used as an indicator of varying attention or, more generally, quality of information processing in action binding tasks (Haggard et al., 2002). “Outcome timing variability” and “trial type” showed no significant main effects or interaction effects on the standard deviation of time judgement errors across subjects (mean ± SD: 65.5 ± 19.2 ms (“action only” trials in “fixed outcome timing” blocks), 66.8 ± 22.5 ms (“action + tone” trials in “fixed outcome timing” blocks), 70.1 ± 24.4 ms (“action only” trials in “variable outcome timing” blocks), 65.4 ± 26.5 ms (“action + tone” trials in “variable outcome timing” blocks). Furthermore, there was no significant effect of “outcome timing variability” on pitch discrimination sensitivity (mean *d*′: 2.52 (“fixed outcome timing”) and 2.34 (“variable outcome timing”), *t*_14_ = 1.22, *p* = .24).

For completeness, we report the shifts observed for “action + tone” trials in “variable outcome timing” blocks in which the action – outcome delay (and, therefore, bottom-up drive) differed from “fixed outcome timing” blocks. Compared to “action only” trials in “variable outcome timing” blocks, subjective action time in “action + tone” trials for which the action – outcome delay was 50 ms or 450 ms did not show the shift towards the outcome observed for the action – outcome delay of 250 ms. When the tone followed the button press after 50 ms, there was even a significant shift away from the outcome when compared to “action only” trials (mean ± SD in “action + tone” trials: −3.5 ± 18.2 ms; *t*_15_ = 2.92, *p* = .011) while there was no significant change due to outcome presentation 450 ms after the button press (mean ± SD in “action + tone” trials: 12.1 ± 22.9 ms; *t*_15_ = 0.81, *p* = .43).

## 4 Discussion

We demonstrate that an essential aspect of action awareness – the experienced time of an action – can be re-constructed by a context-dependent process at the time of the action’s outcome. Specifically, we observe this re-construction of action awareness when the action is performed in a context of uncertain outcome timing. This context-dependency suggests a model-based, top-down re-constructive process that shifts action awareness based on previously experienced action – outcome intervals. If the reconstructive process had been directly triggered by the perceptual trace of the tone, and was independent of the action – outcome delay experienced on other trials, then it could be interpreted as a bottom-up backwards influence. This would be similar to the effects of backwards masking on conscious perception (Werner, 1935), and would not track changes in the time relation of an action and a subsequent stimulus. Context-dependency makes this bottom-up account unlikely.

The majority of previous studies on retrospective action binding have compared conditions that differed in bottom-up drive (“action only” vs. “action + tone” conditions) (Moore & Haggard, 2008; but see Moore et al., 2009). Therefore, these studies cannot exclude the possibility that a retrospective shift of subjective action time is due to bottom-up interference with action awareness or, at a lower level, with the integration of the visual information of the Libet clock. Despite the possibility of such bottom-up driven interference of perceptual codes with action awareness that is unrelated to a representation of the action – outcome association, retrospective action binding has been considered to reflect implicit post-hoc inference on agency (Moore & Haggard, 2008; Voss et al., 2010).

To avoid the difference in bottom-up drive present in previous studies, and to test whether re-construction of action awareness indeed tracks the temporal relation between the action and its outcome, we compared “action only” and “action + tone” trials in two contexts. These contexts differed with respect to temporal outcome variability while the structure and timing of trial events (of interest) was identical across contexts. The interaction between “trial type” (outcome presented vs. omitted) and “outcome timing variability” we observed suggests that a retrospective shift of subjective action time in our study results from an integration of sensory and contextual information on an action – outcome association. This context-dependency speaks in favour of a top-down process underlying retrospective action binding, which integrates prior knowledge and sensory re-afferences.

Compared to previous studies on retrospective binding, notably by Moore and Haggard (2008), our study design introduces an additional aspect of outcome uncertainty. Moore and Haggard exclusively manipulated the probability of outcome *occurrence* while we also varied outcome *timing* with respect to the action. Our current results can neither exclude a retrospective binding component in “action + tone” trials when the action – outcome delay is fixed, nor, indeed, in “action only” trials, as the omission of an expected outcome in these trials may well constitute a sensory event (Sutton, Tueting, Zubin, & John, 1967) and thus alter action awareness retrospectively. In addition, since the probability of outcome occurrence was high across both operant conditions, we cannot exclude a prospective shift of subjective action time from the baseline condition to the operant conditions. However, our key finding is an enhancement of action binding in “variable outcome timing” blocks from “action only” trials to “action + tone” trials, i.e., a clear retrospective process that follows outcome presentation. In addition, we find no change in action binding upon outcome omission as a function of temporal predictability. This may suggest that, unlike retrospective binding, prospective action binding is more closely associated with the expected *occurrence* than the expected *timing* of an outcome. However, this negative result should be interpreted with caution.

Our finding points to a stronger re-constructive component when outcome occurrence *and* timing are uncertain. This suggests that sensory evidence of the action – outcome association is weighed flexibly, depending on the temporal predictability of the outcome. As outcome timing becomes less predictable, sensory evidence is given a relatively stronger weight than outcome prediction and is used for a retrospective adjustment of action awareness.

An interesting question is whether temporal outcome uncertainty interacts with specific action – outcome delays during this re-constructive process. In theory, enhancement of retrospective binding by temporal uncertainty could depend on a central estimate of the distribution of previously experienced action – outcome delays. Alternatively, enhanced retrospective binding could reflect time constants inherent to sensorimotor processing or an absolute time range over which an action is *a priori* expected to cause a sensory event. The present study cannot distinguish between these alternatives. Here, we find enhanced retrospective binding for an action – outcome interval of 250 ms but not for trials with shorter or longer delays (50 ms and 450 ms). Trials with shorter and longer action – outcome delays were necessary to build a context of temporal uncertainty in the “variable outcome timing” condition. These trials had no counterparts in the “fixed outcome timing” condition which could have confirmed that subjective action time is, in principle, shifted under operant conditions for these delays. Indeed, a recent study found no action binding at a fixed action – outcome delay of 50 ms at all (Walsh & Haggard, 2013). We know of no previous study that has systematically examined action binding across a broad range of action-outcome delays (whereas outcome (tone) binding (Haggard et al., 2002) and compound measures, which do not distinguish between tone binding and action binding (e.g. Humphreys & Buehner, 2009), have been studied across a broad range of action-outcome intervals). Because of this, we are cautious to draw strong conclusions regarding action binding in trials with an action – outcome delay of 50 ms or 450 ms in the “variable outcome timing” condition.

In principle, our study design confounds two aspects of a contingent action – outcome association, namely temporal predictability and temporal control (Hughes, Desantis, & Waszak, 2012). Recent evidence supports the view that temporal binding reflects (voluntary) temporal control rather than action-independent temporal predictability (Desantis, Hughes, & Waszak, 2012; Haggard et al., 2002). Temporal binding is specific for action – stimulus associations and does not occur when one stimulus predicts another. By design, we cannot unambiguously attribute our finding of retrospective action binding to either temporal control or temporal predictability. This question goes beyond the purpose of this study, which was to distinguish between bottom-up interference and model-based inference in retrospective binding, and requires attention in future studies.

A contextual influence on retrospective action awareness has been demonstrated in one previous study (Moore et al., 2009). This study varied the probability of a sensory event in trials in which no motor action was performed, thereby modulating the action – outcome contingency in a contextual manner, as in our study. The authors reported retrospective binding under conditions of high, but not low action – outcome contingency, i.e., evidence for an interaction between sensory evidence and context that is compatible with our own finding. Interestingly, we find enhanced retrospective binding when the outcome is not fully determined by the preceding action due to temporal uncertainty. This finding is in agreement with studies that describe retrospective action binding as a post-hoc adjustment of the subjective time of an action upon unpredictable outcome occurrence. From a theoretical perspective, this adjustment seems unnecessary when outcome predictability is high, i.e., when internal models are accurate, as in our “fixed outcome timing” condition. Under conditions of low temporal predictability, however, an internal model of outcome timing can, in principle, be used to evaluate an experienced action-outcome association retrospectively in relation to prior knowledge about the distribution of action-outcome intervals. The probability and timing of a sensory event given an action may affect re-construction of action awareness in a way that is distinct from the effects of contingency as shown by Moore et al. (2009).

In summary, we demonstrate that retrospective action awareness results from a model-based, top-down process, rather than from bottom-up interference with action awareness. This re-constructive process integrates contextual and sensory information to infer on the action – outcome association and may contribute to establishing action – outcome associations under conditions of temporal outcome uncertainty.
